# How to Achieve Better Results Using PASS-Based Virtual Screening: Case Study for Kinase Inhibitors

**DOI:** 10.3389/fchem.2018.00133

**Published:** 2018-04-26

**Authors:** Pavel V. Pogodin, Alexey A. Lagunin, Anastasia V. Rudik, Dmitry A. Filimonov, Dmitry S. Druzhilovskiy, Mark C. Nicklaus, Vladimir V. Poroikov

**Affiliations:** ^1^Department of Bioinformatics, Institute of Biomedical Chemistry, Moscow, Russia; ^2^Department of Bioinformatics, Medical-Biological Department, Pirogov Russian National Research Medical University, Moscow, Russia; ^3^Computer-Aided Drug Design Group, Chemical Biology Laboratory, Center for Cancer Research, National Cancer Institute, NIH, NCI-Frederick, Frederick, MD, United States

**Keywords:** ChEMBL, bioactivity data, kinase inhibitors, SAR, PASS, virtual screening, classification, SAVI

## Abstract

Discovery of new pharmaceutical substances is currently boosted by the possibility of utilization of the Synthetically Accessible Virtual Inventory (SAVI) library, which includes about 283 million molecules, each annotated with a proposed synthetic one-step route from commercially available starting materials. The SAVI database is well-suited for ligand-based methods of virtual screening to select molecules for experimental testing. In this study, we compare the performance of three approaches for the analysis of structure-activity relationships that differ in their criteria for selecting of “active” and “inactive” compounds included in the training sets. PASS (Prediction of Activity Spectra for Substances), which is based on a modified Naïve Bayes algorithm, was applied since it had been shown to be robust and to provide good predictions of many biological activities based on just the structural formula of a compound even if the information in the training set is incomplete. We used different subsets of kinase inhibitors for this case study because many data are currently available on this important class of drug-like molecules. Based on the subsets of kinase inhibitors extracted from the ChEMBL 20 database we performed the PASS training, and then applied the model to ChEMBL 23 compounds not yet present in ChEMBL 20 to identify novel kinase inhibitors. As one may expect, the best prediction accuracy was obtained if only the experimentally confirmed active and inactive compounds for distinct kinases in the training procedure were used. However, for some kinases, reasonable results were obtained even if we used merged training sets, in which we designated as inactives the compounds not tested against the particular kinase. Thus, depending on the availability of data for a particular biological activity, one may choose the first or the second approach for creating ligand-based computational tools to achieve the best possible results in virtual screening.

## Introduction

Discovery of novel pharmaceutical agents with improved safety and efficacy is the perpetual task of medicinal chemistry (Pammolli et al., 2011). In addition to the traditional methods of chemical synthesis and pharmacological studies of various drug-like substances, in recent years substantial attention has been paid to the analysis of the general chemical-biological space (Lipinski and Hopkins, 2004; Baell and Holloway, 2010; Bon and Waldmann, 2010; López-Vallejo et al., 2012; Deng et al., 2013; Medina-Franco et al., 2013; Buonfiglio et al., 2015; Rodriguez-Esteban, 2016; Horvath et al., 2017). Such approaches significantly increase the diversity of the studied chemical libraries as well as the chances to identify the pharmaceutical agents interacting with multiple molecular targets and causing additive or synergistic desired pharmacological action (Sidorov et al., 2015; Lauria et al., 2016).

Nowadays, available chemical libraries can be divided into four categories: (1) databases containing information about structure and properties of publicly disclosed chemical compounds, e.g., PubChem (Li et al., 2010; Wang Y. et al., 2014) and ChEMBL (Bento et al., 2014); (2) databases containing information about structure of commercially available chemical samples, e.g., ZINC (Sterling and Irwin, 2015); (3) databases of virtually generated structures comprehensively covering the particular chemical space, e.g., GDB-17 (Ruddigkeit et al., 2012); (4) databases of virtually generated, synthetically accessible, structures with data on starting materials and proposed synthetic routes, e.g., SAVI (Synthetically Accessible Virtual Inventory) (Pevzner et al., 2017). Although GDB-17 is one of the largest^1^ currently known libraries of chemical structures containing 166.4 billion possible molecules up to 17 atoms of C, N, O, S, and halogen, SAVI looks more attractive for utilization in drug discovery because of the synthesability of its molecules. Furthermore, it was shown (Pevzner et al., 2017) that the overlap between the 93 million structures from PubChem with the 238 million SAVI database is only about 0.03%. Thus, SAVI represents a significant previously unexploited reservoir of novel structures, presumably useful for drug discovery.

To reveal the hidden pharmacological potential of the synthesizable molecules from SAVI, computer-aided virtual screening could be applied (Jorgensen, 2004; Nettles et al., 2006; Bajorath, 2014; Fujita and Winkler, 2016; Lee et al., 2016). Although structure-based methods are widely used now, ligand-based methods have important advantages (Leelananda and Lindert, 2016). In several case studies, machine learning approaches were shown to surpass the performance of both chemical similarity assessment and reverse docking (Anusevicius et al., 2015; Druzhilovskiy et al., 2016; Murtazalieva et al., 2017).

Thus, it is reasonable to analyze the probable biological activity of SAVI molecules using our computer program PASS that recently received high marks: “One of the earliest and most widely used examples of data-mining target elucidation is the continuously curated and expanded Prediction of Activity Spectra for Substances (PASS) software, which was assimilated from the bioactivites of more than 270,000 compound-ligand pairs” (Mervin et al., 2015). The PASS development started more than 25 years ago (Poroikov et al., 1993; Filimonov et al., 1995), and during this time its performance has continuously and significantly improved. PASS in its 2017 version predicts over 7,000 kinds of biological activity with an average accuracy of 94% based on the analysis of structure-activity relationships for more than 1 million known biologically active compounds.

Initially, in the PASS training set a molecule is designated as “active” if reliable information about some biological activity is found in a authoritative source (publication in a peer-reviewed journal, record in curated database, etc.); otherwise, it is designated as “(conditionally) inactive.” This would seem to be a reasonable approach as it has been found that if the same set of chemical compounds is studied against the same molecular target in the three different assays, only 35% of active compounds completely coincided (Lipinski and Hopkins, 2004).

Since no one chemical compound has been tested for all known biological activities, this may appear to be the incorrect designation in some cases. However, it has been shown that PASS provides reasonable estimates of structure-activity relationships despite the incompleteness of information in the training set on both chemical structures and biological activities, due to the robustness of the Naïve Bayes approach in general (Rish, 2001; Rennie et al., 2003) and the MNA descriptors and the biological activity representation used in PASS in particular (Poroikov et al., 2000).

Quantitative data on structure and activity of many chemical compounds freely available from ChEMBL and PubChem databases allow one to consider alternative approaches for creating training sets that may improve the performance of machine learning methods. Such possibilities were recently considered in several studies (Heikamp and Bajorath, 2013; Smusz et al., 2013; Kurczab et al., 2014; Afzal et al., 2015; Mervin et al., 2015).

In this work we evaluated the PASS performance in virtual screening for kinase inhibitors with training performed using three approaches, which differ with respect to what compounds were selected as inactives: (1) only experimentally validated (“true”) inactives; (2) combining true and conditionally inactives; (3) only conditionally inactives. The first and second approaches have the drawback that they require enough data on true inactives.

These training strategies are both related to the multi-label classification (Tsoumakas et al., 2010; Cherman et al., 2011; Afzal et al., 2015) and positive unlabeled learning (Kilic and Tan, 2012), because one and the same classifying object may simultaneously belong to several categories [have multiple labels, i.e., inhibit more than one kinase (Martin et al., 2011) in our case study] and the problem of inactives' selection may be solved using more than one method. In contrary to various approaches of inactives' selection described by the authors (Kilic and Tan, 2012), we used only straightforward approaches, since in chemoinformatics we are forced to deal with extremely sparse data about ligand-protein interactions and, thus, introduction of data about target-to-target relations during the training may lead to strong overfitting.

The kinases were chosen for this study because of the strong family ties among kinases that manifest themselves through common structural features and predispose kinase inhibitors to polypharmacological action (Knight et al., 2010; Gani et al., 2015; Sidorov et al., 2015). Thus, the aforementioned differences in the training set formation may lead to visible changes in the virtual screening performance. Although this class of protein targets has a privileged place in contemporary drug discovery and there are thus many compounds that have been assayed against several or even numerous kinases (Fedorov et al., 2007; Gao et al., 2013; Christmann-Franck et al., 2016; Elkins et al., 2016), multitarget action is found only for a small and diverse subset of the whole chemical-biological space (Jasial et al., 2016).

Therefore kinases and their inhibitors represent an interesting and challenging case that provides useful insights into the influence of the multitarget action of chemical compounds on the success of virtual screening studies (Merget et al., 2017). Moreover, since the multitarget action is by definition an attribute of thoroughly studied compounds, such as FDA-approved drugs (Law et al., 2014), whereas most known compounds are not thoroughly studied, our results may be extrapolated to the target classes (Barelier et al., 2015; Munoz, 2017) less extensively studied compared to kinases, to help achieve better results in virtual screening of a huge chemical library.

## Materials and methods

### Brief description of PASS

PASS (Filimonov et al., 2014) is a computer program for analysis of structure-activity relationships (SAR) that allows users to perform ligand-based virtual screening for ligands of multiple targets and/or compounds with desired biological activities (Abdou et al., 2017; James and Ramanathan, 2017; Stasevych et al., 2017; Yildirim et al., 2017). Structures of chemical compounds are represented in PASS as a set of 2D atom-centric substructural descriptors called MNA (Multilevel Neighborhoods of Atoms). It was previously shown that MNA descriptors are suitable for implementation in a wide range of qualitative (classification) SAR studies and reflect structural features important for ligand–target interactions (Filimonov et al., 1999). PASS predicts biological activity profiles for chemical compounds in standardized representation: uncharged, single-component, containing at least three carbon atoms, with molecular mass not exceeding 1,250 Da. The majority of drug-like molecules fulfill these conditions and clipping of the non-drug-like compounds allows us to avoid dealing with non-specific and atypical biological activities. The mathematical approach of PASS is based on a naïve Bayes classifier and its particular realization in PASS has been previously described in detail elsewhere (Filimonov et al., 2014).

The result of PASS prediction is a list of probable biological activities arranged in descending order of P_a_-P_i_ values, where P_a_ is the probability of belonging to the class of “actives,” while P_i_ is the probability of belonging to the class of “inactives”. By default, P_a_-P_i_ > 0 is considered as the cutoff for discrimination between “active” and “inactive” molecules. The result of PASS-based virtual screening for a chemical library is the list of molecules predicted as “actives”; and these could be recommended for biological testing.

### Training and test datasets

#### Data acquisition

Every dataset used in this study was formed based on the data contained in the ChEMBL database. We chose ChEMBL because this is one of the largest freely available sources of experimental bioactivity data, its data are well-organized and documented, they are easy to acquire (via graphical web interface or API), and easy to manipulate by setting-up a local version of the database. We used the list of protein kinases and their IDs that is available via the ChEMBL web interface by browsing targets by assigned protein classes to select the subset of targets for this case study.

The training set of chemical structures and activities of chemical compounds tested for inhibition of protein kinases was extracted from the 20th version of the ChEMBL database. The ChEMBL SQL-format file dump (dump itself and instructions are available from here: ftp://ftp.ebi.ac.uk/pub/databases/chembl/ChEMBLdb/releases/chembl_20/)was handled in MySQL, SQL queries and PHP scripts were used to manipulate the data and write them to external SD files. Basic validation and comparison of the virtual screening performance were executed using 5-fold cross-validation.

The external test sets contained data from the up-to-date 23rd version of ChEMBL on structures and activities not present in ChEMBL 20 (ftp://ftp.ebi.ac.uk/pub/databases/chembl/ChEMBLdb/releases/chembl_23/). ChEMBL 23 contains 1 154 583 new data on activities, among which we searched for those related to the targets involved in our study using the following procedure:

- We extracted the list of pairs of identifiers of chemical compounds and biological targets from both ChEMBL 20 and 23.
- Intersections between the lists (identical pairs) were excluded.
- We used the remaining pairs to perform virtual screening and compare the results obtained using the three aforementioned approaches.

#### Data preparation

It is known that some noise and various contradictions are stored in, and migrate from one source of bioactivity data to another, along with correct records (Kramer and Lewis, 2012; Kalliokoski et al., 2013; Tiikkainen et al., 2013; Papadatos et al., 2015). Thus, it is necessary to filter the data before using them in order to eliminate incorrect data and records that are inconsistent with the goal of the virtual screening study (Fourches et al., 2016). To achieve this goal, we used the procedures described in our previous work (Pogodin et al., 2015) with slight differences, designed to reflect the peculiarities of the targets selected for this study.

##### Training data preparation

First, chemical structures were filtered to eliminate incorrect molecular representations and to provide PASS with unambiguous (in the given feature space of MNA descriptors) examples for training and validation. We used an in-house command-line utility (SDF-check) to check structures for PASS compatibility and remove unsuitable ones. In addition to this, we identified structures having different ChEMBL IDs, but the same sets of MNA-descriptors, i.e., equivalent structures. We treated such structures as a single one. Thus, data on their activities were joined together, and all structures except first one encountered were deleted from the set.

After the filtering and preparation of the structures, data on bioactivities were processed to remove unreliable and inconsistent data points. In this study we used the following endpoints: K_i_, K_d_, IC_50_, Potency—assessed as concentration of compound that induces the given response; Activity, Inhibition and Residual Activity—assessed as response of the kinase, induced by the given concentration of the compound. In addition to duplicates and incomplete records, the following data were excluded:

- Records related to mutated kinases. Kinases with mutations can have different sensitivity to inhibitors, i.e., quantitatively they may really represent distinct targets, but in general they do not have their own ChEMBL IDs. This fact, taken together with the large number of different mutated forms, makes use of such data difficult and redundant in the context of this study.
- Records related to the (Q)SAR and docking studies of kinase inhibitory activity without experimental validation of the results provided. Unfortunately, calculated values of kinase inhibitory activities may be found in databases along with those measured experimentally, since data are collected automatically using text mining procedures. Even the subsequent curation of the collected data does not allow removal of all questionable data due to the large amount of diverse data. We searched for such records and excluded them, since semi-supervised learning (Rosenberg et al., 2007) was not planned to be studied in this work.
- Records where Activity, Inhibition, or Residual Activity values were provided for a concentration other than 1 μM.
- Records where activation of kinases was provided instead of inhibition.
- Records related to non-standard types of action: inhibition of unphosphorylated kinases (without ATP or prior to ATP addition), allosteric and covalent inhibition, substrate-competitive inhibition (PPI, [protein-protein interaction]). Such cases were excluded since structure-activity relationships for inhibitors of such types may differ (Cortés-Ciriano et al., 2015; Bosc et al., 2017) from the ATP-competitive inhibitors, which represent the majority of known inhibitors.
- Records where kinase inhibitory activities were assigned to the compounds on the basis of their influence on the phenotype of various cells and tissues. Biochemical studies are better suited for the purpose of our study, since they allow to precisely measure the effect of a chemical compound against the particular protein kinase.
- Data on inhibition of non-human kinases were also excluded.

Measurements assessed as response of the kinase (Activity, Inhibition, Residual Activity), induced by the given concentration of a chemical compound were transformed to Inhibition for convenience. The problem with the “Activity” records is their ambiguity. Such records may mean both Inhibition and Residual Activity. We clarified the meaning based on the content of the assay description field. Residual Activity and Inhibition are unambiguously connected (Residual Activity = 100 − Inhibition) and it was easier for us to deal with only one (Inhibition) type of measurement.

Records on the bioactivities were filtered semi-automatically, utilizing the content of the “Description” field from the “Assays” table. Distinct “Description” fields were reviewed and, in the cases of detection of ambiguous data, analogous records were found using suitable set of words or regular expressions. Thus, identified suspicious entries were inspected using the original publications and deleted, if the suspicions were confirmed.

To improve the validation reliability, we included in the study only those kinases that had at least 100 actives and 100 inactives (determined at the concentration 1 μM). These limitations also help with the creation of accurate classifiers, which may be used for their primary purpose: to search for novel kinase inhibitors. Attempts to balance sets in terms of actives to inactives ratio were not conducted, not in the least because the assessment of the difference in the quality of classifiers built on the training data with a different ratio of actives to inactives was of interest, since two of the studied approaches for the training set creation may be considered as a method to fight skewed training data distribution (Rennie et al., 2003).

After the filtering of the bioactivities, different measurements of the inhibitory activity were used to create overall qualitative assessments for each compound designating it as active or inactive against the particular kinase. As it was mentioned earlier, we had different types of data on activities in our set for some compounds. Within these types (percentage of kinase inhibition and compound concentration producing response), median values were calculated in case a given kinase-ligand pair had multiple assessments. If concentrations of compound were available and it was less than or equal to 1 μM, we designated it as active against the particular kinase. In cases where data on concentration of compound were absent we designated it as active if inhibition of the particular kinase produced by this compound was greater than or equal to 50%. Otherwise the compound was designated as inactive.

**Figure 1:**
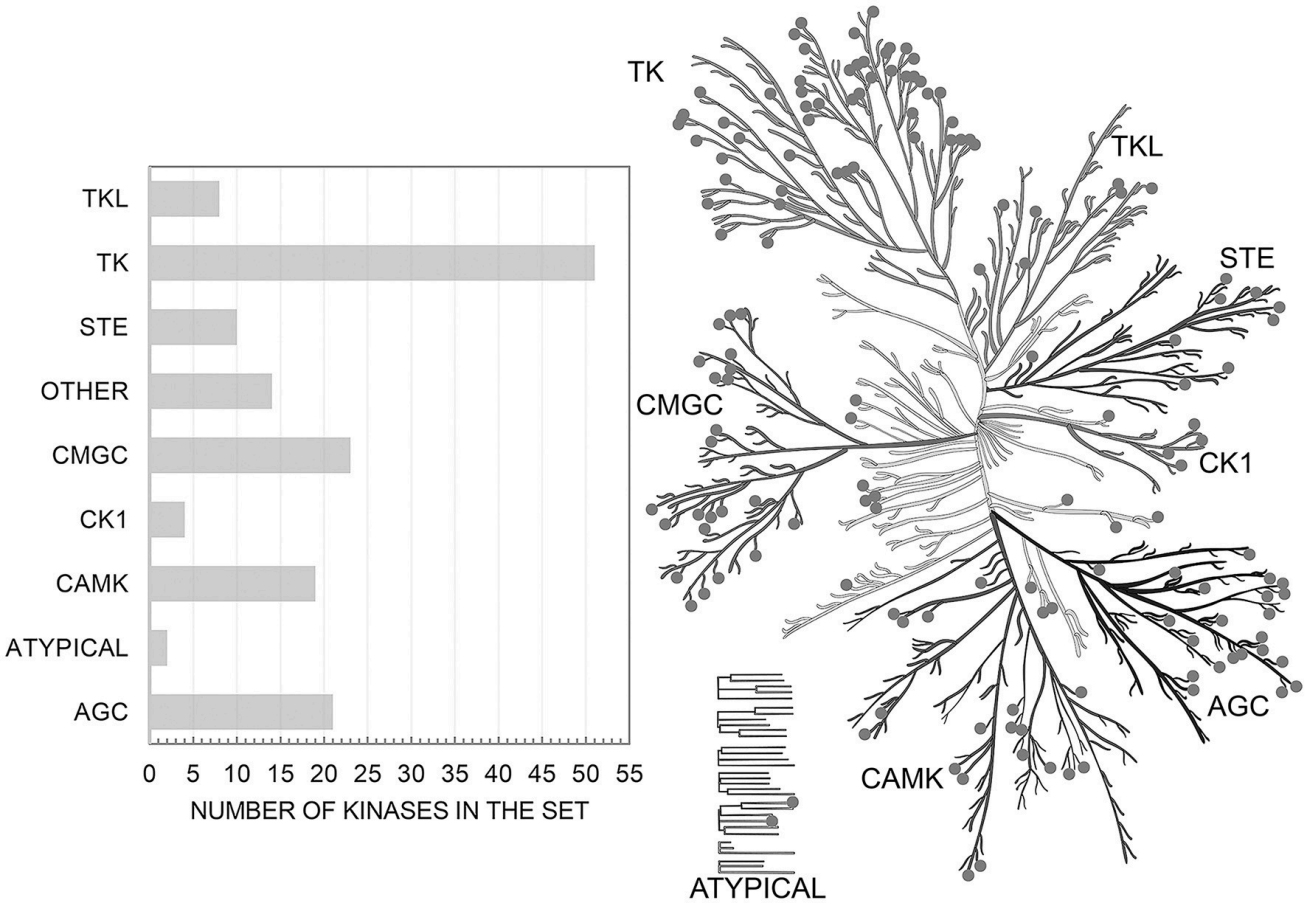
Distribution of targets from the set over the human kinome tree. Illustration reproduced courtesy of Cell Signaling Technology, Inc. (www.cellsignal.com).

Initially we extracted from ChEMBL 458 863 records on kinase inhibition. After the completion of the all procedures described above we were left with 173 275 data points on kinase inhibitors evaluated relative to the cut-off value of 1 μM (62 309 on true actives and 110 966 on true inactives at given cut-off). These data characterize interactions of 55 162 compounds with one or more of 152 human protein kinases selected for this study. These kinases represent all major families of human kinases. Our data cover a significant portion of the human kinome and allow one to search for inhibitors for all kinase families (Figure 1).

##### External test data preparation

Preparation of the data for external test set was performed in the same way as for the training set data, except for the following differences:

- Chemical data were not filtered, since done automatically by PASS.
- Potency was excluded from the list of the relevant activity types, since the majority of such activity records do not contain any data in the field “standard_relation.”
- Activity was excluded from the list of the relevant activity types, since the majority of such activity records do not fulfill the requirement of absence of mutations, and/or compound concentration is not relevant to the selected cut-off.
- No minimum numbers for actives and inactives were imposed.

In total, we were able to identify 81 563 new activities against the kinases involved in this study in the 23rd version of ChEMBL. After filtering, 35 317 activities describing the action of 23 004 compounds against kinases remained.

##### Training set formation approaches

**Figure 2:**
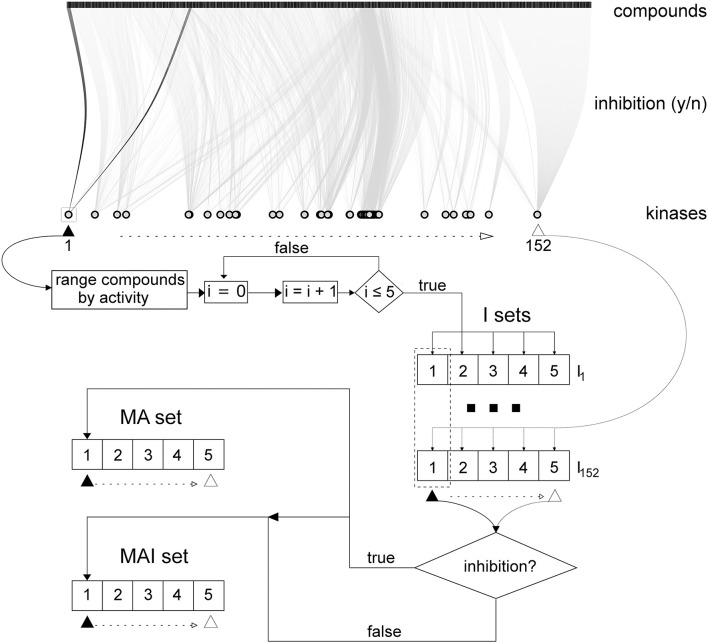
Scheme describing how the training sets were created in accordance with the different approaches. Initial data on kinase inhibitors are represented via bipartite graph using all kinases and subset (~ 6 000) of tested compounds. Initially for 152 kinases 5x152 I-sets had been created; then, MA- and MAI-set were created as described in section Merged Actives and Inactives Set (MAI-set) and Merged Actives Set (MA-set).

Filtered training set data on kinase inhibitors were stored in the local MySQL database and used to create three different training sets described below and presented in Figure 2. In addition, each training set was divided into the five non-overlapping and equivalent subsets for subsequent stratified 5-fold cross-validation (5-f CV).

##### Individual sets (I-sets)

The tested compounds for each kinase were sorted from the most active to the most inactive and, in this order, they were written to the five SD files: the first compound in the rank was placed into the first subset, the second compound into the second subset, the fifth compound into the fifth subset, the sixth then again into the first subset and so on; until each compound was placed into the each corresponding subset. The subsets were created in this way to be equivalent in terms of the total number of compounds and similar to each other in the degree of inhibitory activity of the placed compounds.

##### Merged actives and inactives set (MAI-set)

Then, we merged the first, second etc. subsets for each of the 152 kinases. If identical compounds were found in different subsets, only the structural formula was retained with all its kinase inhibiting activity data. As a result, we obtained 5 combined MAI-subsets, which were equivalent to the I-subsets because these subsets contained the same active compounds.

##### Merged actives set (MA-set)

This set was created in the same manner as MAI-set, but the true inactives were excluded.

### Quality metrics

We used the following metrics to evaluate the results of our ligand-based virtual screening of kinase inhibitors:

(1) SENSITIVITY(RECALL)=TP/(TP+FN)

(2) SPECIFICITY=TN/(TN+FP)

(3) $$BALANCED\;ACCURACY=\;\frac{1}{2}{\;}^{*}(\frac{TP}{TP+FN}+\;\frac{TN}{TN+FP})$$

(4) PRECISION=TP/(TP+FP)

(5) $$F1=2*\frac{PRECISION*RECALL}{PRECISION+RECALL}$$

(6) $$\begin{array}{l} ROCAUC=P\left(Rank_{active_{i}}<Rank_{inactive_{i}}\right)\\ \text{ in Uniform distribution} \end{array}$$

(7) $$\begin{array}{l} BEDROC=P\left(Rank_{active_{i}}<Rank_{inactive_{i}}\right)\\ \text{ in exponential Probability Density}\\ \text{Function }(\text{PDF})\text{ with parameter }\alpha ,\text{ IF }\alpha^{*}\text{Ra }<<\text{ 1} \end{array}$$

Metrics (1–6) are appropriate for the evaluation of the performance of the classification procedure, which determines the upper limits of the virtual screening quality under condition where every compound predicted as active is screened experimentally.

Boltzmann-Enhanced Discrimination of Receiver Operating Characteristic (BEDROC) (Truchon and Bayly, 2007) (Equation 7) represents the adaptation of ROC AUC metric to conditions under which detection of maximal number of TPs in a certain top fraction of the set is more important than general recognition. Thus, it is designed to evaluate the early detection rate, i.e., to assess the quality of virtual screening under the limitation that it is possible to evaluate experimentally only small fraction of top rated compounds from the whole library. Parameter α in the BEDROC AUC is inversely related to the size of the top fraction that will contribute to 80% of the score value while the other 20% will come from the assessment of the remaining part of the set. Values of α that were used in this study, and the corresponding top fractions of the sets, are given in Table 1.

**Table 1:** Values of BEDROC parameter α and corresponding top fractions of sets.

**Top fraction**	**BEDROC α**	**Actives rate**	α ^*^ actives rate
1.00%	160.9	0.001	0.161
3.00%	53		0.054
5.00%	32.2		0.032
8.00%	20		0.020
10.00%	16.1		0.016
16.10%	10		0.010
20.00%	8		0.008

### Performance assessments

#### Stratified 5-fold cross-validation

The training data had been divided into the five subsets in such a way that the average numbers of actives and inactives were approximately equal in all subsets (Refaeilzadeh et al., 2009). Four subsets from each set were used for the training, while one subset was used as the external test set. This procedure was repeated five times; each time a different subset was used as the external test set. The main differences from the standard 5-fold CV were:

-Corresponding individual subsets were always used as test, regardless of set type utilized for training.
- Compounds were placed into the subsets not on a random basis, but according to their degree of inhibitory activity.

**Figure 3:**
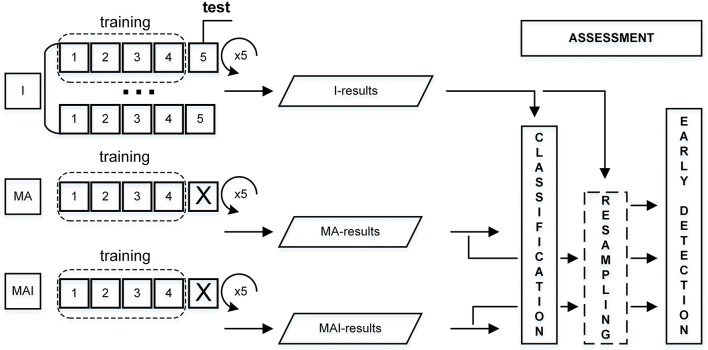
Scheme for performance evaluation. I-subset was always used as a test set, thus corresponding MA- and MAI-subsets were just excluded from training.

The overall scheme for performance evaluation is given in Figure 3.

Such validation procedure provides reliable quality assessments for classifiers, since every compound in the test sets had experimental test results against a particular kinase. Besides, such an approach provides the conditions for comparison that are close to those observed in real research projects when one tries to find novel activity for a compound already included in the training set with some other activities. Such situations occur in drug repurposing projects or in *in silico* toxicological studies (Wang Y. J. et al., 2014).

The results of the predictions were assessed using the metrics described in the Materials and Methods section. Unfortunately, at least one of them, BEDROC, may suffer from saturation. To avoid this, the ration of actives to inactives for a set (Ra in Formula 7) must be low enough to fulfill the condition given in Formula 7.

The condition of low fraction of actives in the set seems acceptable and reasonable in the context of high throughput screening, which typically provides a number of hits below 5% (Murray and Wigglesworth, 2017). However, the data on kinase inhibitors from our set do not fulfill this condition. Thus, the saturation effect on BEDROC was expected to affect the results of our study. To avoid BEDROC saturation, we implemented the procedure of random sampling with replacement as realized in R package mlr (Bischl et al., 2016) applied to the prediction results. We undersampled the portions of actives and oversampled the portions of inactives for each kinase. Factors to under- and oversample actives and inactives were chosen in such a way that numbers of actives and inactives in the resampled set became equal to approximately 60 and 60 000, respectively (Formulae 8, 9). Thus, we maintained the same actives rate in the resampled sets, which was chosen to be approximately 0.001. This rate is low enough to calculate BEDROC values for each α level selected for this study without the risk of saturation.

(8) Factor actives=60/Number of actives

(9) Factor inactives=60000/Number of inactives

The resampling procedure was repeated 5 000 times for each type of sets and each kinase to achieve statistical significance in the subsequent assessment of differences between the results. BEDROC values were calculated on the resampled data using the R package enrichVS (http://cran.r-project.org/web/packages/enrichvs/index.html) for each resampled set. ROC AUC was also calculated using the R package pROC (Robin et al., 2011). To increase the speed of obtaining resampling results, we performed calculations in parallel mode using R package “parallel” (https://stat.ethz.ch/R-manual/R-devel/library/parallel/doc/parallel.pdf). Values of the classification quality metrics achieved in cross-validation and training set composition could be found in Supplementary Table 1.

#### Virtual screening of the external test set

Prepared data from 23rd version of ChEMBL was used for forming the test sets according to the procedure used for preparation of the training I-sets. During the external validation (Chen et al., 2012) with these sets we calculated BEDROC values for the resampled prediction results. Values of the classification quality metrics achieved in external validation and training set composition could be found in Supplementary Table 2.

### Comparison of the results obtained using different training approaches

The Tukey honest significant difference (HSD) test was used along with the analysis of variance to compare the quality of the created PASS classifiers based on the different types of training sets. These quality parameters include BEDROC for the resampled results; sensitivity, specificity, balanced accuracy, precision, F1 score and ROC AUC for the original results. The analysis was performed at a *P*-value < 0.05 using the functions “aov” and “TukeyHSD” from the R standard library. This provides the ranked lists for three PASS classifiers, which allows one to evaluate their performance.

## Results

### Stratified 5-fold cross-validation

**Figure 4:**
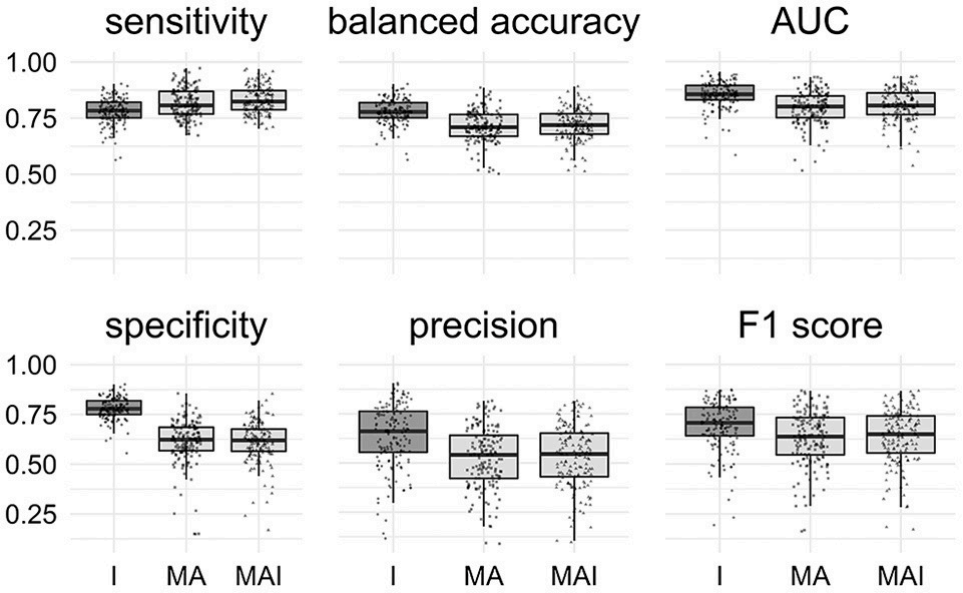
Comparison of the results obtained using different types of sets. Results that are significantly different (*P*-value < 0.05) from one another are colored by distinct shades of gray. Results were obtained using stratified 5-f CV. Points correspond to the results achieved for the distinct kinases, shape of the points corresponds to the type of the training set.

All classification metrics values averaged over all kinases except the sensitivity values were slightly higher for the results achieved by classifiers trained on I-sets. Statistical analysis indicates that results obtained using the I-sets differ significantly from those obtained with the MA and MAI sets (Figure 4). The results of classifiers trained on the MA- and MAI-sets do not differ at the given level of significance from each other.

**Figure 5:**
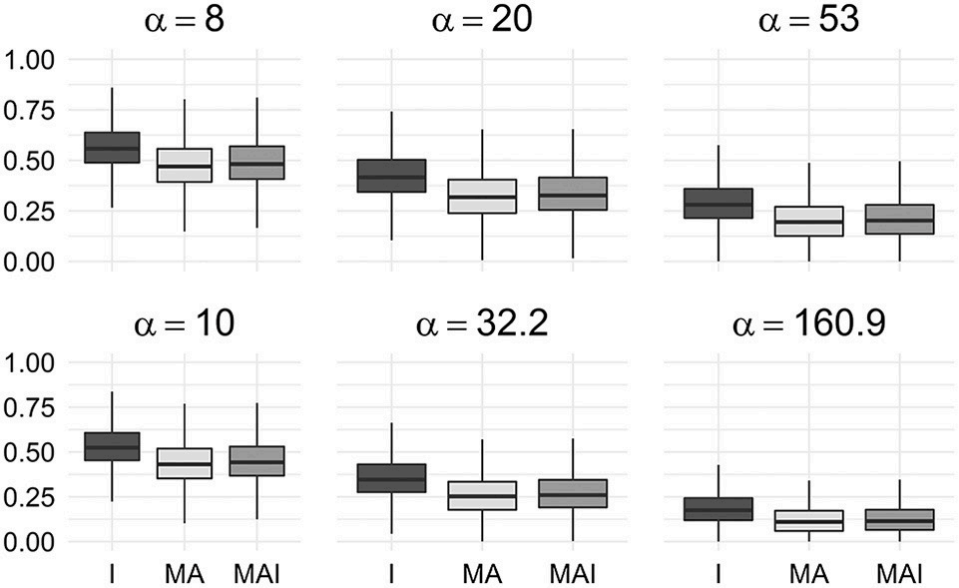
Comparison of BEDROC values of resampled results for different kinases. In this case results obtained using different type of training sets were significantly different from each other (*P*-value < 0.05) for any value of α. Results were obtained using 5-f CV.

We used the resampled results to calculate values of BEDROC at different degrees of early recognition of TP (via varying values of α). These values were grouped according to the types of sets used for the training, and then averaged over the kinases in a manner similar to the way the original results were obtained. Statistical analysis of these data shows that classifiers trained on I-sets significantly outperform classifiers trained on MAI-sets and those, in turn, outperform classifiers trained on MA-sets (Figure 5) for any α value used in the study.

**Figure 6:**
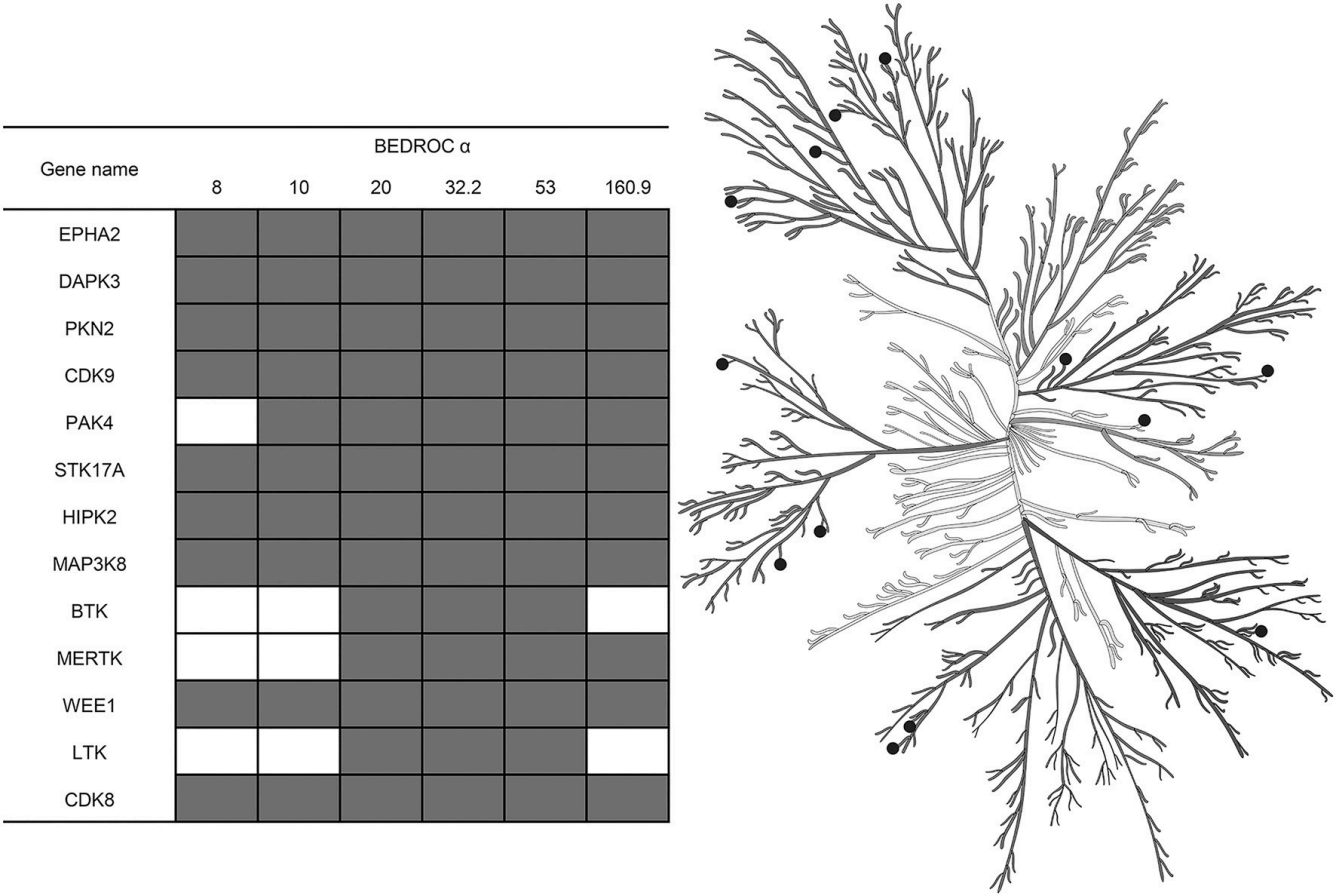
Kinases for which inhibitors may be found at top ranks using MA- or MAI-training sets, according to the evaluation based on the resampling technique (*P*-value < 0.05). Empty cells correspond to the cases where I-sets still perform better. Illustration reproduced courtesy of Cell Signaling Technology, Inc. (www.cellsignal.com).

Also, using the resampled results, we were able not only to compare different approaches for the training by averaging values of the selected metrics across kinases, but to select the most adequate approach for each kinase individually. This was because after the resampling procedure repeated 5,000 times, we had enough data points to estimate the statistical significance. Such estimation was performed as follows: at the level of the *P*-value chosen earlier, less than 0.05, we found that for most of the kinases the best approach for training is to use I-sets; nonetheless, for some kinases it is better to use MA- or MAI-sets (Figure 6) according to our evaluation. In total, we depicted 13 kinases for which the classifiers trained using MA- or MAI-sets performed better in early recognition of TP at at least three levels of α.

### Virtual screening of external test set

Since we did not impose any limitations on the number of actives and inactives in our external test set, we were not able to calculate values for all the metrics for each kinase. We excluded such kinases before averaging the values of the classification metrics across the different training approaches, thus only results for 128 kinases were compared.

**Figure 7:**
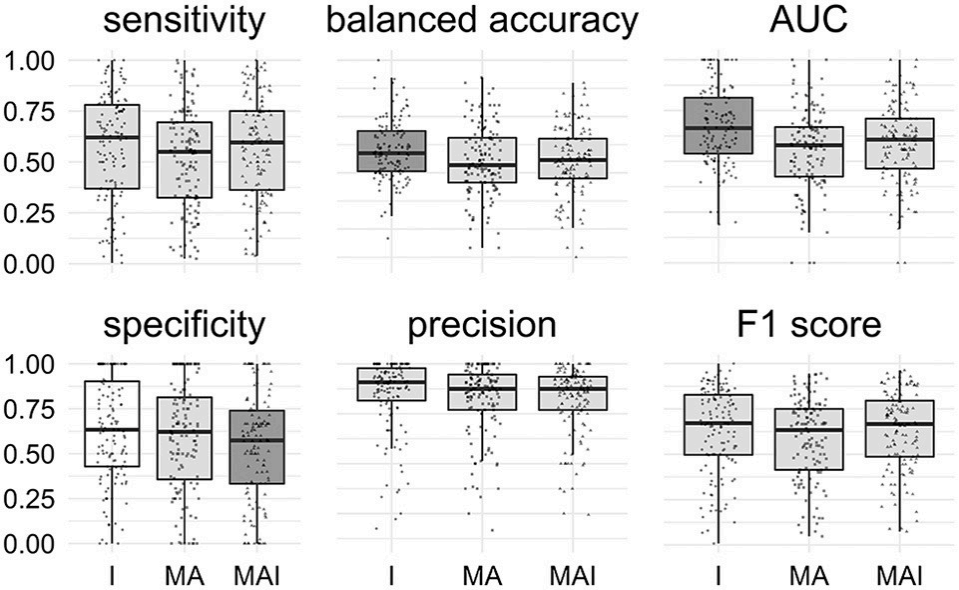
Comparison of the results obtained using different types of sets. Results that are significantly different (*P*-value < 0.05) from one another are colored in distinct shades of gray. Results were obtained using external test set. Points correspond to the results achieved for the distinct kinases, the shape of the points corresponds to the type of the training set.

The main conclusions of the comparison of Specificity, Balanced Accuracy, and AUC values are similar to those obtained using 5-f CV: The training approach I provided significantly better results than those introducing conditionally inactives (MA and MAI). No significant difference for the other metrics was found (Figure 7).

To compare the earliness of actives detection achieved using different training approaches, we resampled results of the inhibitory activity prediction for each kinase and calculate BEDROC values. In this part of the study only results related to kinases having at least 20 actives and 20 inactives in the external test set were included. This restriction was imposed to exclude the influence of extreme cases, where only few actives and inactives exist. Despite the introduced restrictions, we were forced to change the resampling protocol in some cases; if the kinase had less than 60 actives, we used an oversampling procedure instead of undersampling to make sure we had 60 actives.

**Figure 8:**
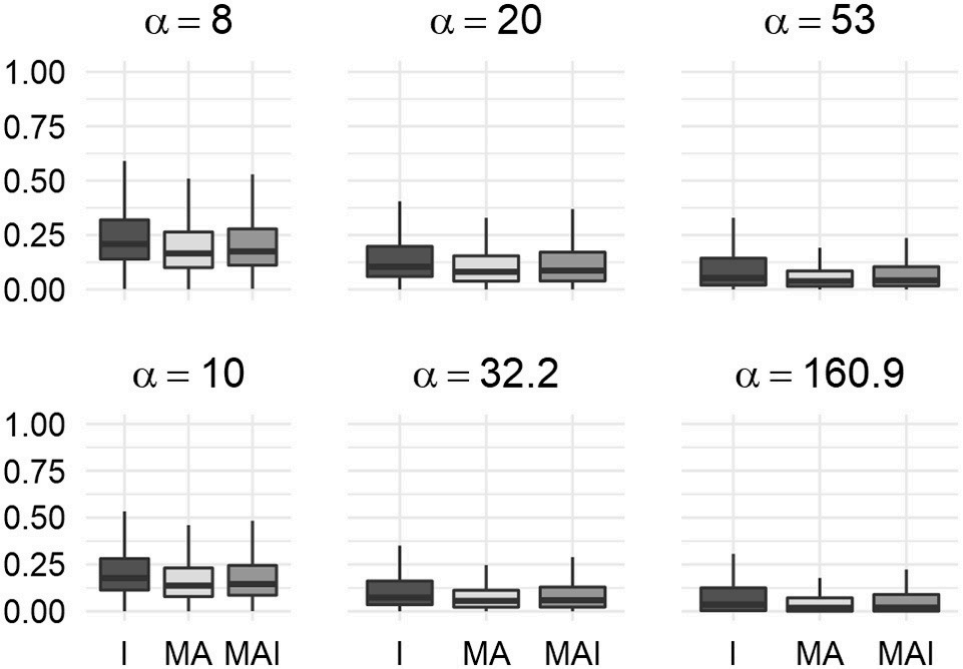
Comparison of BEDROC values of the resampled results for distinct kinases. In this case results obtained using different type of training sets were significantly different from each other (*P*-value < 0.05) for any value of α. Results were obtained using the external test set.

The main result of the comparison of BEDROC values was concordant to those obtained using 5-f CV: at each value of the criterion α, training using I-sets led to the better results than training performed using MA- or MAI-set, while MAI-sets outperformed MA-sets (Figure 8).

### Correlations between the values of metrics and actives to inactives ratio in the sets

We also analyzed the behavior of the employed accuracy metrics for different actives/inactives ratios, to be sure that they give an unbiased picture.

Values of Precision and F1-score were found to show correlations with the actives to inactives ratio in the test sets. Thus, we conclude that sets' imbalance affects Precision and F1-score values, while the other metrics are significantly more robust (see Supplementary Figure 1), especially AUC and Balanced Accuracy.

### Applicability domain estimation

To estimate the applicability domain, we calculated the values of the classification quality metrics for those cases where compounds had a certain number of new MNA-descriptors not found in the training set. In this case we merged the results over all kinases to obtain sufficient numbers of data points.

**Figure 9:**
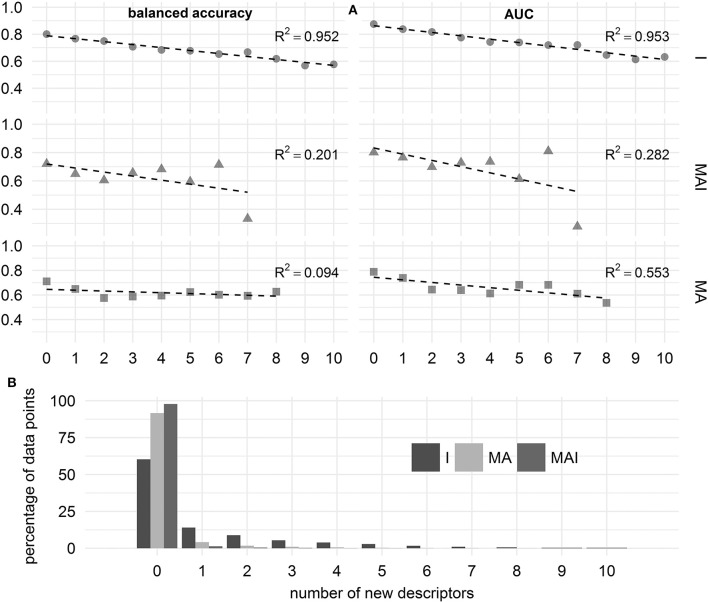
Influence of the number of new descriptors in the structure of chemical compound on the quality of prediction. **(A)** Balanced accuracy and AUC assessed using different types of training sets. **(B)** Percentage of data points characterizing by the different number of new descriptors.

We showed that in the case of the results achieved using I-sets for training, the performance of the classifiers decreases linearly with increasing number of new MNA descriptors. In contrast to this, for the results achieved using MA- and MAI-sets for training, we were unable to find a strong dependence between the number of new MNA descriptors and the performance of the classifiers. Still, these results should be treated with caution, since the percentage of data points involved in this assessment decreases drastically with increasing number of new MNA descriptors, especially for the classifiers built using MAI- and MA-training sets (see Figure 9).

In the case of the classifiers built using I-sets for training we can judge that the applicability domain includes those compounds which have 4 or fewer new MNA descriptors, since the average balanced accuracy and AUC exceeded 0.7.

## Discussion

In contrast to the many contemporary studies in the field of the virtual screening, in this work no decoys (Irwin, 2008) were used to assess the enrichment achieved in virtual screening of large datasets. Instead, validation and subsequent comparison of the different training approaches were performed using only experimentally tested compounds, both actives and inactives. Today, due to the constant growth of available computational resources and amount of bioactivity data, it is possible to do this using 5-f CV and true external test sets. Moreover, since negative influence of the conditionally inactive compounds involved in training was shown, this makes us wonder: if conditionally inactives can do harm during training, are decoys good for testing? The exact answer is not known yet, but the risk of reaching wrong conclusions may be mitigated by using resampling-based approaches in parallel with, or instead of, decoys.

Our study represents a quantitative assessment of the trade-off between the initial requirements on the training data and the quality of PASS-based virtual screening. We have shown that the most efficient training approach for the ligand-based virtual screening system is to use the true actives and inactives for each target. This approach outperformed those where conditionally inactive compounds were introduced, in both classification quality and earliness of the detection. Moreover, in this case we observe a strong dependence of the performance depending on the number of new descriptors in the structures of the test compounds.

**Figure 10:**
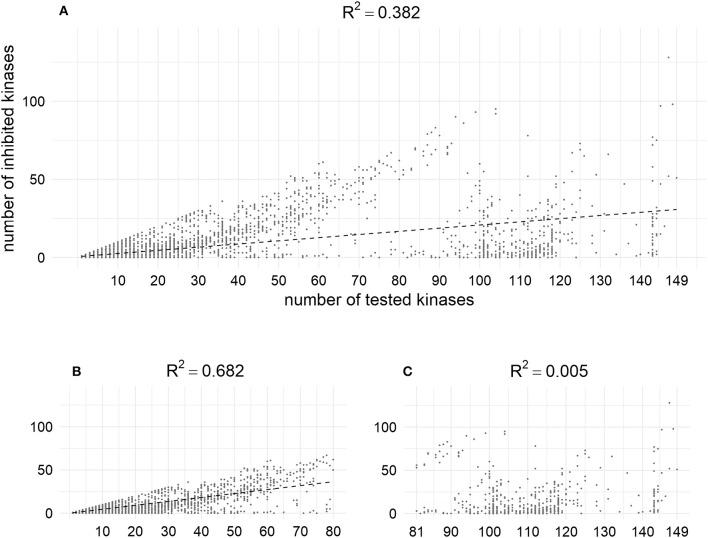
Positive dependence between the number of tested kinases and number of inhibited kinases in the training set; **(A)** Relation for the whole set (weak); **(B)** Relation for the fraction of compounds tested against less than 80 kinases (moderate); **(C)** Relation for the fraction of compounds tested against more than 80 kinases (not observed). X–coordinates correspond to the number of chemical compounds against which compounds were tested, Y-coordinates corresponds to the amount of kinase against which compounds were found to be active at given cut-off (1 μM).

According to the analysis of the data from our training set, the higher the number of kinases for which compounds are tested, the more activities are found. Thus, using MA and MAI sets for training, some unknown actives could be treated as conditionally inactives (Figure 10). This may shed some light onto the problem of promiscuity of kinase inhibitors, which are often discussed as polypharmacological drugs. However, analysis of the content of bioactivity databases such as ChEMBL has shown that the average degree of promiscuity of such compounds is not so high (Hu et al., 2014). According to our results there is no contradiction between these points of view: kinase inhibitors tend to show promiscuity, but at the moment most of them have been studied against only a rather limited number of kinases.

Nevertheless, using MA and MAI approaches, it is possible to achieve good virtual screening results too, despite the softer requirements on the amount and quality of the training data. These approaches may be implemented in cases when only few active compounds are known, even in the absence of inactives, which helps expand the druggable target space and find new modes of action for existing molecular targets.

From this perspective it is surprising that we also found 13 kinases for which virtual screening may be performed more efficiently using training approaches introducing conditionally inactive compounds. This means that using machine learning it is easier to distinguish between inhibitors of these kinases and compounds tested against other kinases, than between their inhibitors and inactives at the given concentration cut-off. This fact can possibly be explained by the systematical shift in compounds selection for testing against these kinases. Also, it may indicate the importance of small structural changes in related targets leading to larger changes in inhibitor potency, since these 13 kinases are diverse, they belong to different families represented in our set and, in the case of other members of their families, introduction of the conditionally inactive compounds leads to the observed negative consequences. Thus, we show that virtual screening performance may benefit from the introduction of conditionally inactive compounds if these compounds are unfamiliar to the main target. Unfortunately, this knowledge is risky to apply to achieve better results in ligand-based virtual screening, since our knowledge on target-target relations mediated by common ligands are generally based on sparse training sets.

We obtained rather good results of both external (quasi prospective) and cross-validation. However, in case of data on kinase inhibitors extracted from ChEMBL, one initially deals with the pre-selected compounds studied in the appropriate biological activity area, which provides good predictivity, particularly using the approach based on individual sub-sets.

Big libraries like SAVI contain diverse and previously not investigated chemical structures, including compounds other than those possessing known ligand-related target signatures (Sidorov et al., 2015). To achieve the best predictivity for such library, it seems reasonable to make pre-selection with the standard PASS approach using conditionally inactive compounds. As we already mentioned above, PASS provides satisfactory results of prediction despite the incompleteness of data in the training set (Poroikov et al., 2000). Moreover, in this work, we showed that classifiers created using the merged training sets did not exhibit the significant dependence between the prediction quality and the number of new MNA descriptors contained in the predicted chemical structures.

Consequently, we propose two-steps procedure to analyze the big and diverse chemical libraries. At the first step, pre-selection is performed using the general classifier that took into account the conditionally inactives. At the second step, one may more thoroughly discriminate between the active hits and putatively inactive structures using the specific classifier that is based only on the real actives and inactives.

## Conclusions

In this study, we compared the performance of three approaches for the analysis of structure-activity relationships that differ in their criteria for selecting “active” and “inactive” compounds for the training sets. We used the program PASS to build classifiers based on different subsets of kinase inhibitors extracted from ChEMBL 20 (for training and 5f-CV) and ChEMBL 23 (for external, quasi-prospective validation). The highest classification and early recognition quality was obtained by using individual training sets for each kinase containing only experimental data. Nevertheless, other training strategies can provide acceptable results even in the absence of data on known inactives, which is often the case with the novel targets (Russ and Lampel, 2005; Nguyen et al., 2017). We assessed the applicability domain of our classifiers: while classifiers trained using individual sets expose strong dependence of the prediction quality on the predicted compounds' novelty, training strategies employing merged sets are much less sensitive to the novelty of predicted compounds.

Taken together these findings allow us to suggest that one can benefit most from using combinations of different training strategies when exploring huge chemical libraries containing diverse structures of unexplored chemical compounds.

## Author contributions

All authors listed have made a substantial, direct and intellectual contribution to the work, and approved it for publication.

### Conflict of interest statement

The authors declare that the research was conducted in the absence of any commercial or financial relationships that could be construed as a potential conflict of interest.

## Footnotes

^1^The Danish biopharmaceutical company Nuevolution announced that it had created a library of 40 trillion unique molecules (C&EN, 2017, 95: 28–33); however, the web site (https://nuevolution.com/technology) states that the company enables DNA encoded synthesis of billions of chemically diverse drug-like small molecule compounds.
