# Supplementary figures and images for: How to Achieve Better Results Using PASS-Based Virtual Screening: Case Study for Kinase Inhibitors
Source: Front Chem. 2018 Apr 26;6:133. doi: 10.3389/fchem.2018.00133 (PMC5935003; doi:10.3389/fchem.2018.00133)

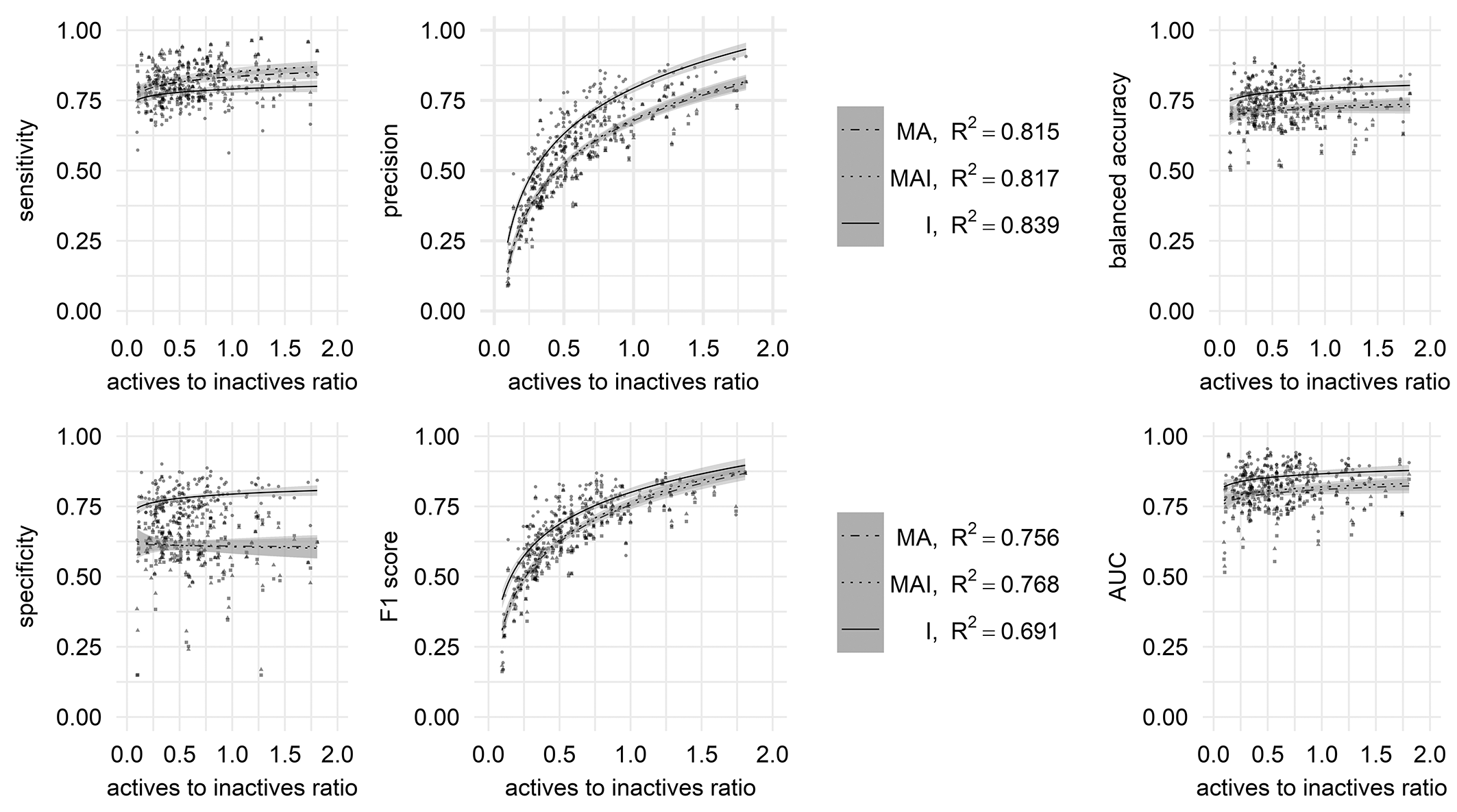

Supplement: Supplementary file 3 [file Image_1.TIF]
