# Supplementary material for: How to Achieve Better Results Using PASS-Based Virtual Screening: Case Study for Kinase Inhibitors
Source: Front Chem. 2018 Apr 26;6:133. doi: 10.3389/fchem.2018.00133 (PMC5935003; doi:10.3389/fchem.2018.00133)
Supplement: Supplementary file 4 [file Data_Sheet_1.docx]

Supplementary Material

HOW TO ACHIEVE BETTER RESULTS USING LIGAND-BASED VIRTUAL SCREENING: CASE STUDY FOR KINASE INHIBITORS

Pavel V. Pogodin^1^, Alexey A. Lagunin^1,2^, Anastasia V. Rudik^1^, Dmitry A. Filimonov^1^, Dmitry D. Druzhilovskiy^1^, Mark C. Nicklaus^3^, Vladimir V. Poroikov^1*^

*** Correspondence:**

Prof. Vladimir V. Poroikov
vladimir.poroikov@ibmc.msk.ru

**Supplementary Figures:**

**Supplementary Figure 1.** Correlations between values of metrics and actives to inactives ratio in the sets. R2 values are given for F1 score and Precision cases only, since in other cases significant correlation is absent.

**Supplementary Tables:**

**Supplementary Table 1.** Values of the classification quality metrics achieved in cross-validation and training set composition. File consists of three lists corresponding to the different types of the training sets.

**Supplementary Table 2.** Values of the classification quality metrics achieved in external validation and test sets composition. File consists of three lists corresponding to the different types of the training sets.
